# Morphological and Functional Changes of Meibomian Glands in Pediatric and Adult Patients with Allergic Conjunctivitis

**DOI:** 10.3390/jcm11051427

**Published:** 2022-03-04

**Authors:** Yuqing Wu, Hao Jiang, Xujiao Zhou, Zimeng Zhai, Pei Yang, Shuyun Zhou, Hao Gu, Jianjiang Xu, Jiaxu Hong

**Affiliations:** 1 Department of Ophthalmology, Eye and ENT Hospital, Fudan University, Shanghai 200031, China; yokchingwu@163.com (Y.W.); xujiao.zhou@fdeent.org (X.Z.); zimeng_zhai@163.com (Z.Z.); pei_28@163.com (P.Y.); zhoushuyun@sina.com (S.Z.); jianjiang-xu@163.com (J.X.); 2 Department of Ophthalmology, The Affiliated Hospital of Guizhou Medical University, Guiyang 550004, China; kidd5jh@sina.com (H.J.); guhao@gmc.edu.cu (H.G.)

**Keywords:** allergic conjunctivitis, meibomian glands, child, dry eye, tear film

## Abstract

Allergic conjunctivitis (AC) is one of the most common ocular disorders in clinical practice and is associated with meibomian gland dysfunction. This study aimed to explore the morphological and functional changes of meibomian glands (MGs) in pediatric and adult patients with AC and to analyze their potential predictors. In our prospective, observational cohort study, a total of 59 patients with AC were enrolled, with 30 patients aged ≤16 years in the pediatric group and 29 patients in the adult group. All patients underwent examinations at baseline and last visit when the complete resolution of conjunctival papillae was identified. An automatic MG analyzer was used to measure the morphological and functional parameters of MGs, including their area ratio (GA), tortuosity index (TI), and signal index (SI). Two groups were comparable at baseline in terms of characteristics and MG parameters (*p* > 0.05). The morphological (length, square, and GA) and functional MG parameters (SI) of AC patients significantly improved in the pediatric group after treatment (all *p* < 0.05), but not in the adult group. The change in the GA correlated with age, sex, GA, TI, and SI at baseline (all *p* < 0.05). Age (*p* = 0.001) and GA (*p* < 0.001) at baseline were predictors of an improvement in the GA of MGs. The findings showed that the structure and function of MGs in pediatric patients with AC seem to improve after the conjunctival papillae disappear, but not in adult patients.

## 1. Introduction

Allergic conjunctivitis (AC) is one of the most common ocular disorders in clinical practice [1], with a 28% incidence in China [2], a nearly 25% incidence in children in Europe [3], and between 20% and 30% incidence in the United States [4]. The major symptoms of AC are itching, swelling, and tearing, and in some severe cases, photophobia and pain could develop due to the involvement of the cornea [5,6]. Patients suffering from AC have a lower health-related quality of life and impaired socioeconomic status [7,8,9,10].

Ocular allergy contributes to tear film hyperosmolarity, ocular surface inflammation, and damage, all of which are key mechanisms in the vicious cycle dry eye disease (DED) [11]. TFOS DEWSII has identified AC as a probable risk factor for DED [12]. The prevalence of DED in pediatric patients with AC ranges from 12% to 97.5% [13,14]. MGD is a major long-term complication of AC [15], and patients with atopic keratoconjunctivitis (AKC) have been reported to be affected by recurrent MGD [16,17,18]. Previous studies have described the relationship between the structure of the AC and the meibomian glands (MGs). Arita et al. revealed a greater distortion of the meibomian gland duct in patients with contact lens-related AC and perennial AC [19,20]. Ibrahim et al. suggested that patients with AKC present a worse change in MG morphological parameters compared to patients with obstructive MGD and the controls [18]. The inflammation response and continuous mechanical stress induced by chronic eye rubbing in AC are presumed to be associated with the onset of meibomian gland dysfunction (MGD) [20,21]. The occlusion of MGs in AC has been reported to be related with T cell-mediated [22] and aggregated neutrophil extracellular traps [23]. The potential reversibility of the MG morphology has been controversial for a long time [24,25,26], and much research in recent years has focused on this issue. Yin et al. and Hura et al. reported an improvement in gland morphology in adults with MGD after treatment [27,28,29]. However, to the best of our knowledge, no previous studies have investigated whether and how meibomian glands change in AC patients, especially in the pediatric population.

## 2. Materials and Methods

### 2.1. Subjects

This prospective study was approved by the Institutional Review Board of the Eye and ENT Hospital of Fudan University (EENTIRB-20190301) and adhered to the tenets of the Declaration of Helsinki. All subjects who presented to the Ophthalmology Department of the Eye and ENT Hospital of Fudan University from August 2019 to September 2021 were enrolled and gave informed consent prior to their inclusion in the study. Inclusion criteria were patients with clinical diagnosis of AC based on symptoms of redness, itching, frequent blinking, photophobia, and clinical findings of palpebral conjunctiva papillae, conjunctival hyperemia, or corneal epithelial exfoliation. Exclusion criteria were children who could not cooperate with examinations and patients with blepharitis, obvious eyelid or ocular surface disorders, intraocular diseases, contact lens wear, a history of eye surgery, and systemic or ocular diseases that would interfere with tear film production or function [20]. None of the patients had used topical ocular medications, including eye drops and ointments, in the previous 3 months. All the patients were diagnosed with active disease at the time of enrollment, which means that subjects in our study were either newly diagnosed or had relapsed without being medicated for this episode yet [30].

Each patient underwent clinical examinations at baseline and at the last visit when complete resolution of conjunctival papillae was identified. The patients were treated with 0.1% olopatadine (Patanol, S.A. Alcon-Couvreur N.V., Puurs, Belgium) twice daily, or 0.1% tacrolimus (TALYMUS; Senju Pharmaceutical, Co., Ltd., Osaka, Japan) three times daily, or 0.1% fluorometholone eye drops (Flumetholon; Santen, Japan) three times a day. Severe cases were treated with combination therapy, including 0.1% tacrolimus (three times daily) and 0.1% olopatadine (twice daily), or 0.1% fluorometholone eye drops (three times daily) and 0.1% olopatadine (twice daily). Therapeutic treatments were divided into three categories: 1 (monotherapy, the use of topical dual-acting antihistamine/mast-cell stabilizers alone); 2 (monotherapy, the topical use of steroid or immunosuppressive drugs alone); and 3 (combination therapy, the use of dual-acting antihistamine/mast-cell stabilizers and steroid or immunosuppressive drugs). All the examinations were conducted by the same observer during the study. Data used in this study were obtained from the right eye of each subject. If the right eye was excluded from the study, data from the left eye were used.

### 2.2. Clinical Evaluation

The clinical examinations were carried out in the following order, including uncorrected visual acuity, best-corrected visual acuity, a slit-lamp examination of cornea, conjunctiva and anterior segment examination before and after fluorescein drops, corneal fluorescein staining (CFS), tear meniscus height (TMH), noninvasive breakup time (NIBUT), and meibography. CFS was evaluated in five areas (upper, lower, nasal, temporal, and optical diameter) of the cornea and scored from 0 to 3 after the instillation of fluorescein [31]. TMH, NIBUT, and infrared photography of the upper meibomian glands were performed using Keratograph 5M (Oculus, Wetzlar, Germany). The patients were instructed to blink two times and then keep their eyes open to the best of their ability to complete the NIBUT test. The first NIBUT (NIBUT-1st) and the average NIBUT (NIBUT-avg) were calculated automatically by the software [31]. Meibography was scored semiquantitatively as the following grades (Supplementary Figure S1): grade 0 (no loss of meibomian glands), grade 1 (loss of less than one-third of the total area of meibomian glands), grade 2 (loss of between one-third and two-thirds of the total area), and grade 3 (loss of over two-thirds of the total area) [32].

### 2.3. Multi-Parametric Automated Meibomian Gland Analyzer

**Figure 1:**
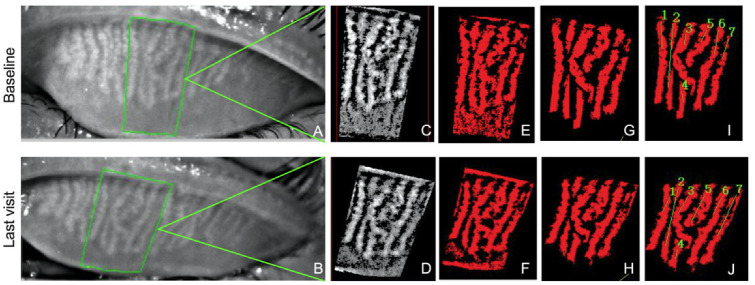
The morphological changes in meibomian glands between baseline (**upper**) and last visit (**lower**). In this study, we chose the same area (a center on the gland of the most significant change, including its left and right three glands) in the original grayscale meibography image for examination to the best of our abilities (**A**,**B**, green outline). The automatic process of reduced random noises, enhanced contrast, and visibility images (**C**,**D**), and automatic generation of the binarized and binary inverted mask image (**E**,**F**). Removal of the tarsal conjunctival area and segmentation of each gland manually (**G**,**H**). Selection of intended analysis meibomian glands (**I**,**J**).

Deng et al. have identified the parameters based on automatic MG analyzer as an invaluable tool for the assessment and evaluation of MG status and objective grading [33]. The meibography score method could only provide semiquantitative records and failed to present subtle changes. Hence, the multi-parametric Meibomian Gland Bioimage Analyzer Version 3 (DMK, Guangzhou, China) was used [33,34]. The analysis of acquired meibography images at baseline and last visit can be divided into five steps: (I) loading dynamic meibomian images of the same patient taken at baseline and at last visit into the software, (II) comparison of the two images and identification of the gland with the most comparable change by evaluating the shape, position, size, atrophy degree of meibomian glands, and other unique characteristics such as palpebral conjunctival blood vessels, (III) manual selection of the region of interest (ROI), which was composed of seven MGs in total, including the comparable and representative, with a center on the gland of the most significant change, including its left and right three glands, respectively (Figure 1A,B), (IV) segmentation of each single gland at a pixel level within the ROI using the delineated tool in the software (Figure 1E–H), and (V) automated measurement and analysis of the morphology and function of each MG in the ROI of the two images, respectively (Figure 1I,J; Supplementary Tables S1 and S2), including the gland diameter (D), gland length (L), gland square (S), gland tortuosity index (TI), gland signal index (SI), and gland area ratio (GA). The gland TI presents the degree of curving and hairpin-loop-like winding changes of the glands. The gland SI, defined as a functional marker, refers to the average image grayscale value of the segmented intact glands divided by the average image grayscale value of the non-gland area [33,34]. The GA is defined as the ratio of the gland area to the ROI area. The exclusion criteria of images included images with incomplete pictured eyelids, low exposure, poor resolution or clarity, and other factors leading to inaccurate comparison such as insufficient eversion of the upper eyelids, the shelter of hair, or eyelashes in front of the meibomian gland. Throughout the manually performed initial comparison, images were magnified and shrunken constantly to ensure the identification of the most comparable ROI. All of the analysis was conducted by a single trained, masked observer (Y.W.).

### 2.4. Statistical Analysis

Data were analyzed using SPSS 20.0 (IBM Corp, Armonk, NY, USA). Continuous data were presented as mean ± standard deviations (SD) and categorical data were presented as n (%). All intergroup variables were analyzed using the Wilcoxon signed-rank test. Continuous variables at baseline and last visit within the group were tested with a paired Wilcoxon signed-rank test. To reduce the potential bias of different GAs between children and adults due to face size and eyelid size, we used the percentage change in GA (%) to evaluate the morphological change in MGs between baseline and last visit. The following numeric calculations were performed in SPSS: the difference in GA (GA at last visit − GA at baseline), the percentage change in GA (%) (the difference in GA/GA at baseline × 100). The prognosis of pediatric and adult patients was tested with the chi-square test. Correlations were analyzed with the Spearman correlation coefficient, respectively. Univariate linear regression was conducted to identify the independent correlation of indexes associated with the percentage change in GA. Multiple linear regression was conducted to verify the predictors of the percentage change in GA, controlling variables that displayed a *p*-value of less than 0.05 in the test of correlation. The statistical significance level was <0.05.

## 3. Results

### 3.1. Characteristics and Clinical Indexes

A total of 59 patients with AC were enrolled. Thirty patients aged ≤16 years were assigned to the pediatric group (age, 7.83 ± 2.67 years, range from 4 to 15; 27 male and 3 female), and the other 29 patients aged >16 (late adolescents and adults) were assigned to the adult group (age, 39.59 ± 12.37 years, range from 17 to 60; 11 male and 18 female). As shown in Table 1, the two groups were comparable at baseline in regard to the characteristics and MG indexes (*p* > 0.05). There were 28 AKC patients (17 in the pediatric group and 11 in the adult group) and 1 VKC patient in the pediatric group. The difference of severe subtypes between groups has no statistical significance (*p* > 0.05), so the comparison of the percentage change in GA in the following analysis is unaffected. The mean treatment duration was 49.79 ± 28.11 days (range from 13 to 119) in the adult group, which was longer than in the pediatric group (34.77 ± 14.12 days, range from 21 to 75; *p* < 0.05). There was a difference in therapeutic treatment between the pediatric and the adult group. All patients in the pediatric group received combined therapy (0.1% tacrolimus 3 times daily plus 0.1% olopatadine 2 times daily), while the patients in the adult group received monotherapy or combined therapy (0.1% fluorometholone eye drops 3 times daily and 0.1% olopatadine twice daily or 0.05% azelastine twice daily and 0.1% cyclosporine twice daily).

### 3.2. Comparison of the Meibomian Gland Morphological and Functional Changes Observed in the Pediatric Group and the Adult Group

**Figure 2:**
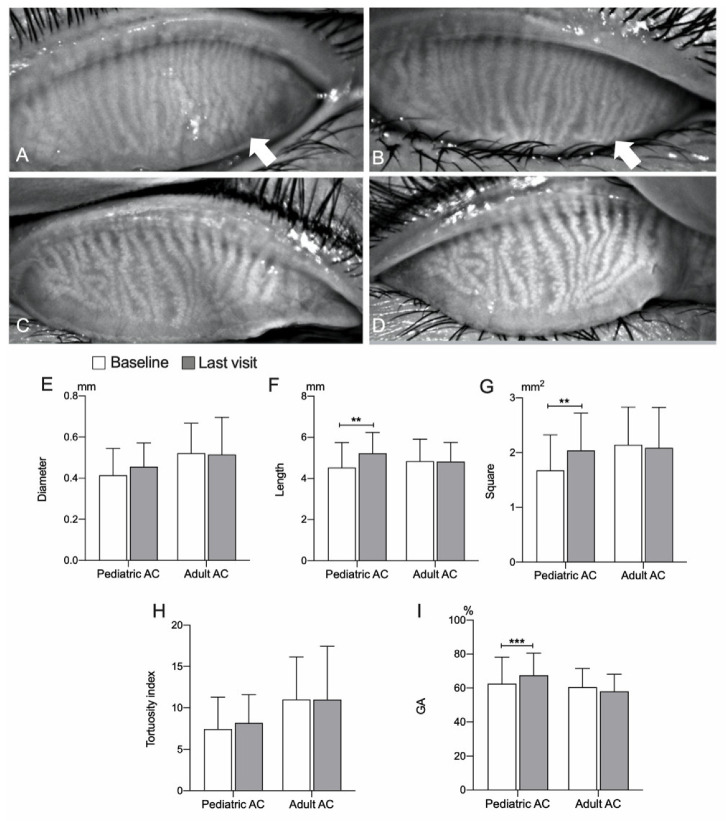
Comparison of meibomian glands morphological parameters between baseline and last visit. Representative images of MGs structural changes (**A**–**D**). Meibography images showed the same upper eyelid of MGs of a 12-year-old patient with AC: (**A**) at baseline (meiboscore = 1) and (**B**) after 22-day treatment (meiboscore = 0). Meibography images showed the same upper eyelid of MGs of a 31-year-old patient with AC, (**C**) at baseline (meiboscore = 1), and (**D**) after 4-month treatment (meiboscore = 1). Note that more significant morphological improvements (white arrow) were observed in pediatric AC patients than in adult AC patients. Diameter, length, square, TI, and GA of pediatric and adult AC patients between baseline and last visit (**E**–**I**). Length, square, and GA were increased significantly at the last visit in pediatric AC patients (**F**,**G**,**I**), whereas no significant change was observed in the adult group. AC, allergic conjunctivitis; TI, tortuosity index; GA, glands area ratio; MGs, meibomian glands. Bars represent mean ± SD. ** *p* < 0.01, *** *p* < 0.001. The paired Wilcoxon signed-rank test.

Figure 2 shows representative cases of MG morphological changes. The pediatric group commonly showed a better improvement in the MG structure than that observed in the adult group. Baseline length (4.53 ± 1.2 mm, range from 0.00 to 6.56 vs. 5.22 ± 1.0 mm, range from 2.98 to 7.53), square (1.68 ± 0.6 mm^2^, range from 0.00 to 3.04 vs. 2.04 ± 0.7 mm^2^, range from 1.07 to 4.01), and GA (62.54 ± 15.7%, range from 0.00 to 85.64 vs. 67.56 ± 13.01%, range from 20.00 to 89.76) significantly improved at the last visit (*p* < 0.01, *p* < 0.01, and *p* < 0.001, respectively) in the pediatric group, but not in the adult group (all *p* > 0.05). Diameter and TI in both groups had no statistical change between baseline and last visit. The percentage change in GA in this study was 2.49 ± 11.67% (range from −35 to 30), 8.17 ± 10.3% (range from −24 to 30) in the pediatric group, and 3.38 ± 10.1% (range from −35 to 12) in the adult group. A total of 86.7% (26/30) patients in the pediatric group presented an improvement in GA and up to 33.3% (10/30) patients showed an increase of more than 10%, whereas only 37.9% (11/29) in the adult group improved (*p* = 0.001, Table 2). The subgroup analysis comparing the adults in regimen 3 with the children showed that there was no statistical difference in the percentage change in GA (*p* > 0.05).

**Figure 3:**
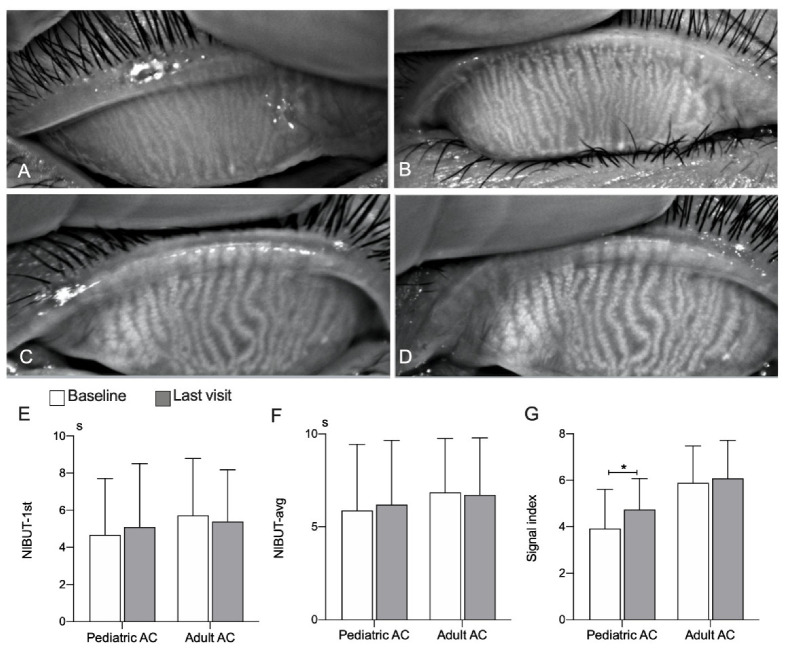
Comparison of meibomian glands functional parameters between baseline and last visit in AC patients. Representative images of functional changes in MGs (**A**–**D**). Meibography images showed the same upper eyelid of MGs of a 5-year-old patient with AC: (**A**) at baseline (SI = 3.23) and (**B**) after 48 days at the last visit (SI = 6.74). Meibography images showed the same upper eyelid of MG of a 54-year-old patient with AC: (**C**) at baseline (SI = 5.84) and (**D**) after 66 days at the last visit (SI =8.07). Note that the clearer and more distinguishable gland figures were observed in the pediatric patients than in adult patients. There was a significant increase in SI at the last visit in pediatric AC patients (**G**), whereas no significant change was observed in adult AC patients (**E,F,G**). AC, allergic conjunctivitis; MGs, meibomian glands; SI, signal index; NIBUT-1st, the first time of noninvasive keratograph tear film breakup; NIBUT-avg, the mean time of noninvasive keratograph tear film breakup. Bars represent mean ± SD. * *p* < 0.05. The paired Wilcoxon signed-rank test.

Figure 3 shows representative cases of MG functional changes, where clearer and more distinguishable gland figures were observed in the pediatric group than in the adult group. Baseline SI (3.92 ± 1.7, range from 0.00 to 8.30 vs. 4.74 ± 1.3, range from 2.29 to 8.27) significantly increased at the last visit (*p* < 0.05) in the pediatric group, whereas there was no difference in SI (5.89 ± 1.6, range from 0.95 to 8.90 vs. 6.09 ± 1.6, range from 0.95 to 8.41) in the adult group (*p* = 0.088). NIBUT-1st and NIBUT-avg in both groups had no statistical change between baseline and last visit.

### 3.3. Factors Related to the Percentage Change in GA

**Figure 4:**
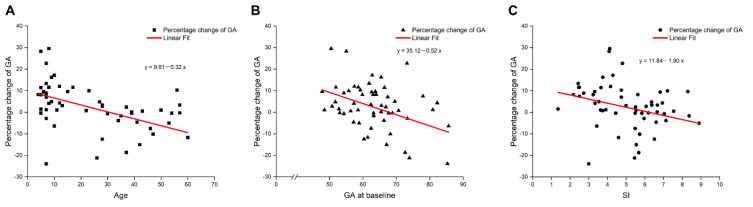
Scatterplots for the correlations between age (**A**), GA at baseline (**B**), SI at baseline (**C**), and the percentage change in GA. Age, GA at baseline, and SI at baseline were significantly correlated with the percentage change in GA (*p* < 0.001, *p* < 0.01, and *p* < 0.05, respectively). The Spearman coefficients were rAge: −0.54, rGA: −0.35. rSI: −0.38. Percentage change in GA (%) (GA at last visit − GA at baseline)/GA at baseline × 100; GA, glands area ratio; SI, signal tortuosity index.

Table 3 shows the results of a Spearman correlation analysis between the percentage change in GA and the possible influencing factors. The results showed five possible influencing factors: age, sex, and MG parameters (GA, TI, and SI at baseline). All of these five variables had a negative correlation with the improvement in percentage change in GA (all *p* < 0.05). Univariate analysis showed increased age, female sex, elevated GA, TI, and SI at baseline to be associated with decreased percentage change in GA (*p* < 0.001, *p* < 0.001, *p* < 0.05, *p* < 0.01, *p* < 0.05, respectively; Supplementary Table S3; Figure 4). Table 4 displays the results from multiple regression analysis for each variable. Five possible influencing factors were included. Among them, age and GA at baseline were the only two variables that were significantly associated with percentage change in GA. The regression coefficients of age and GA at baseline were −0.28 (95%CI [−0.45, −0.12], *p* = 0.001) and −0.55 (95%CI [−0.82, −0.29], *p* < 0.001), respectively. 2013.

## 4. Discussion

To the best of our knowledge, the present study is the first study to investigate MG changes in pediatric and adult patients with AC. Our results showed a significant improvement in MG structure and function in pediatric patients with AC after conjunctival papillae disappear, but not in adult patients. In addition, our study revealed significant correlations between age and MG changes in patients with AC. The automatic analyzer applied in this study facilitated the capture of incremental changes that were almost undetectable via semiquantitive meiboscore [33].

The meibomian gland bioimage analyzer has been proved to be efficient in the assessment of MG morphology and function [33,34]. In this study, we investigated MG structural and functional parameters in AC patients at baseline and the last visit. Our results revealed different changes in MGs after treatment between pediatric and adult AC patients for the first time. Previous noninvasive meibography examinations have revealed that AC affects the structure of MGs [19,20], and confocal microscopy examinations have reported a decrease in the size of MG acinar units and extensive periglandular inflammatory cell infiltration in AKC and vernal keratoconjunctivitis (VKC) patients [18,35,36]. However, very few articles have focused on the changes of MG structure in AC, especially in pediatric patients. The results of the current study showed that there was a 2.49% increase in GA (pediatric group, 8.17%; adult group, −3.38%) in the upper eyelids of patients with AC after the complete resolution of conjunctival papillae, which was in accordance with previous studies on MGD patients. Yin et al. and Arita et al. reported 5% and 4.6% decrease in the dropout rate in the eyelids of all patients with MGD after treatment, respectively [24,27]. Yin et al. also reported that there was a decrease in MG inflammation and an improvement in MG macrostructure, as well as microstructure after treatment in MGD. The reduced MG inflammation resulting from the relief of AC might be the potential mechanism for the recovery of MG structure [27].

As regards MG functional parameters, SI has been suggested as a new parameter in quantifying the optical density of meibum within the ductal system. In this study, there was an increase in SI (3.92 ± 1.7 vs. 4.74 ± 1.3) in the pediatric group, which indicated an improvement in MG function and a higher delivery state of glands, as it has been previously reported that a low-level SI (<4.5) of MGs was identified to harbor a 24.3-fold increased adjusted risk for MGD compared to patients with a medium-level SI (4.5–6.5) [33]. It is possible that the increased delivery state could be a response to compensate for the increased demand of meibum [37,38] and corresponds to the improvement in MG structure [28,39]. Yin et al. showed that the improved expressibility contributed to the reversibility of MG dropout [27]. Further study including an assessment of meibum quality and gland expressibility is needed to explore the change in MG function in AC. Yang et al. reported a significant deterioration in MG function (decreased lipid layer thickness and blinking disorder) in pediatric patients with VKC/AKC [30]. Ibrahim et al. also suggested that the MG dropout and lid margin changes in AKC may cause changes in the lipid layer and a worse tear function [18]. In contrast to previous findings on tear function in AC, NIBUT-1st and NIBUT-avg had no correlation with the change in MG structure in our study. This discrepancy may be explained partially by different patient populations. Here, the majority of patients in our study were less severe forms of AC, whereas patients in previous studies were AKC/VKC.

Interestingly, in the Spearman correlation analysis and multivariate linear regression analysis, age and GA at baseline were statistically correlated with the improvement of GA. Contrary to our conclusions, Yin et al. reported that the reversibility of MG has no correlation with age [27]. The disagreement may be due to the age of patients enrolled in the research. Our study included both pediatric patients and adult patients with AC, while most patients with MGD were adults due to different epidemiological characteristics of diseases. Two studies have shown a correlation between age and meibomian gland loss, with men starting to drop out in their twenties and women starting in their thirties [40,41]. Meanwhile, Parfitt et al. suggested that a loss of meibocyte progenitors is expected to cause glandular atrophy in age-related MGD [42]. Therefore, we hypothesize that the age-related meibomian gland atrophy was responsible for poorer prognosis in adult patients, compared with pediatric patients.

The present study has several limitations. First, different therapeutic treatments were used between two groups, because we rarely used long-term steroid eye drops in children. Our study focused primarily on whether there was a change in MGs after effective treatment, and we defined the last visit as “when the complete resolution of conjunctival papillae after treatment.” Although the treatment methods were different, the desired effectiveness was achieved in both groups. Therefore, we explored the change in MGs on the same basis, the relief of allergic conjunctivitis, which would minimize potential bias of the different treatment methods. Whether this factor would bias our finding requires more investigation. Second, there were more males in the pediatric group, though sex is an important parameter for the morphology of MGs. According to TFOS DEWS II Sex, Gender, and Hormones Report [43], there was a higher incidence of abnormal lid margin and gland dropout in men ≥ 70 years and a higher risk of meibomian gland dysfunction development due to androgen deficiency during menopause. In our study, patients in the pediatric group were under 16 years old, which was unlikely to bias the percentage change in GA significantly. Third, the follow-up time in our study was relatively short and the sample size was small. As a result, our findings may not be generalized to extend to all AC patients. A long-term prospective study with a larger sample size in the future would help us to address this issue. Fourth, in this study, only the upper eyelids were included for the assessment of MG morphology. This is because previous studies have identified that, compared with lower eyelids, the upper eyelids showed a more visible MG structure and higher quality images with less uneven focus [33,44,45]. Finally, we did not analyze the five patients in the pediatric group with allergic rhinitis using nasal sprays in our study. However, previous studies have reported that the effect of intranasal corticosteroids on the ocular surface is limited [46].

## 5. Conclusions

In conclusion, we found that the MG structure and function in pediatric patients with AC seem to improve after the conjunctival papillae disappear, but not in adult patients. This indicated that tear film function should be monitored carefully in adult AC patients even after the disease has been relieved. Whether the partial recovery of MG structure in pediatric patients results from the progenitors or meibocyte proliferation needs more evidence.

## Figures and Tables

**Table 1:** Characteristics and clinical indexes of patients at baseline.

Variables	Children (N = 30)	Range (Min, Max)	Adults (N = 29)	Range (Min, Max)	*p*
Age, year	7.83 ± 2.67	(4, 15)	39.59 ± 12.37	(17, 60)	**<0.001**
Sex, *n* (%)					**<0.001**
Male	27 (90.0)		11 (37.9)		
Female	3 (10.0)		18 (62.1)		
Onset duration, month	4.6 ± 4.2	(2.0, 24.0)	3.9 ± 4.1	(0.3, 24.0)	0.176
Treatment duration, day	34.77 ± 14.12	(21, 75)	49.79 ± 28.11	(13, 119)	**<0.05**
Treatment therapy, *n* (%)					**<0.001**
Regimen 1 ^a^	0 (0.0)		10 (34.5)		
Regimen 2 ^b^	0 (0.0)		11 (37.9)		
Regimen 3 ^c^	30 (100.0)		8 (27.6)		
CFS (0–3)	0.10 ± 0.40	(0, 2)	0.17 ± 0.47	(0, 2)	0.386
Upper MGL (0–3)	0.77 ± 0.68	(0, 3)	1.07 ± 0.65	(0, 3)	0.052
NIBUT-1st, s	4.67 ± 3.05	(1.08, 14.65)	5.73 ± 3.06	(1.21, 14.31)	0.091
NIBUT-avg, s	5.88 ± 3.56	(1.08, 14.65)	6.86 ± 2.90	(1.21, 14.31)	0.110
TMH, mm	0.18 ± 0.03	(0.14, 0.25)	0.20 ± 0.03	(0.15, 0.23)	0.103

Abbreviations: CFS, corneal fluorescein staining; MGL, meibomian gland loss; NIBUT-1st, the first time of noninvasive keratograph tear film break up; NIBUT-avg, the mean time of noninvasive keratograph tear film break up; TMH, tear meniscus height. ^a^: Regimen 1 = 0.1% olopatadine (Patanol, S.A. Alcon-Couvreur N.V., Puurs, Belgium) twice daily. ^b^: Regimen 2 = 0.1% tacrolimus (TALYMUS; Senju Pharmaceutical, Co., Ltd., Osaka, Japan) 3 times daily or 0.1% fluorometholone eye drops (Flumetholon; Santen, Japan) 3 times daily. ^c^: Regimen 3 = 0.1% tacrolimus (three times daily) plus 0.1% olopatadine (twice daily), or 0.1% fluorometholone eye drops (three times daily) plus 0.1% olopatadine (twice daily). Values represent mean ± SD. *p*-values marked in **bold** indicate significance. The Wilcoxon signed-rank test.

**Table 2:** Prognosis of GA in pediatric and adult AC patients.

Group	Total	The Percentage Change in GA (%) ^a^, n (%)							*p*
		Decrease			NC	Improve			
		>10	5–10	<5	0	<5	5–10	>10	
Pediatric	30	3.3	3.3	3.3	3.3	23.3	30.0	33.3	**0.001**
Adult	29	24.1	6.9	13.8	17.2	27.6	3.4	6.9	

Abbreviations: AC, allergic conjunctivitis; NC, no change; GA: gland area ratio. ^a^: The percentage change in GA (%) = (GA at last visit − GA at baseline)/GA at baseline × 100. *p*-values marked in **bold** indicate significance. Chi-square tests.

**Table 3:** Correlation between the percentage change in GA (%) and other variables.

Characteristic at Baseline	Age	Sex ^a^	Onset Duration	Treatment Duration	Upper MGL	GA	TI	SI
r	−0.54	−0.49	0.06	−0.194	0.02	−0.35	−0.51	−0.38
*p*	**<0.001**	**<0.001**	0.656	0.149	0.870	**0.009**	**<0.001**	**0.004**
Difference value of other indexes	ΔTI ^b^		ΔSI		ΔNIBUT-1st		ΔNIBUT-avg	
r	0.19		0.15		0.05		0.14	
*p*	0.150		0.261		0.720		0.287	

Abbreviations: MGL, meibomian gland loss; GA, gland area ratio; TI, tortuosity index; SI, signal index of the glands; NIBUT-1st, the first time of noninvasive keratograph tear film breakup; NIBUT-avg, the mean time of noninvasive keratograph tear film breakup. ^a^: Male = 0, female = 1, set male as control. ^b^: Δ = the difference value between baseline and last visit indexes. *p*-values marked in **bold** indicate significance. The Spearman rank correlation.

**Table 4:** Results of a multiple linear regression analysis examining the impact of relative variables on the percentage change in GA (%).

Predictor	Unstandardized Coefficients Beta	Standardized Coefficients Beta	95% CI		*p*
			Lower	Upper	
Age	−0.28	−0.44	−0.45	−0.12	**0.001**
Sex^a^	−3.55	−0.15	−9.42	2.32	0.230
TI	−0.37	−0.15	−0.92	0.19	0.189
GA	−0.55	−0.42	−0.82	−0.29	**<0.001**
SI	0.24	0.04	−1.46	1.94	0.780

Abbreviations: GA, gland area ratio; TI, tortuosity index; SI, signal index of the glands. ^a^: male = 0, female = 1, set male as control. *p*-values marked in **bold** indicate significance.

## Data Availability

The authors confirm that the data supporting the findings are available from the corresponding author upon reasonable request.

## Supplementary Materials

The following supporting information can be downloaded at: https://www.mdpi.com/article/10.3390/jcm11051427/s1, Figure S1: representative cases of meibomian gland loss grade; Table S1: automatic calculation of parameters in meibomian glands at baseline in the analyzer; Table S2: automatic calculation of parameters in meibomian glands at last visit in the analyzer. Table S3: Univariable analysis examining the impact of relative variables on the percentage change of GA.
