# Supplementary material for: Morphological and Functional Changes of Meibomian Glands in Pediatric and Adult Patients with Allergic Conjunctivitis
Source: J Clin Med. 2022 Mar 4;11(5):1427. doi: 10.3390/jcm11051427 (PMC8911235; doi:10.3390/jcm11051427)
Supplement: Supplementary file 1 [file jcm-11-01427-s001.zip › jcm-1599502-supplementary.pdf]

Supplementary Materials

Supplementary Figures

Supplementary Figure S1.

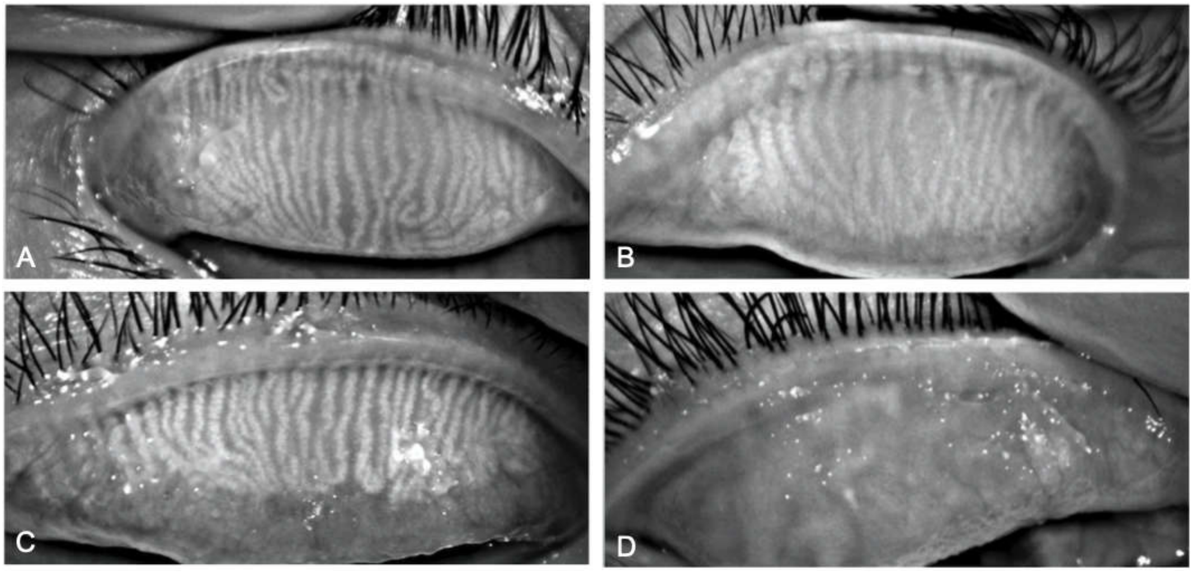

Supplementary Figure S1. Representative cases of each grade of meibomian gland loss, scored using the following grades: grade 0, no loss of meibomian glands (**A**); grade 1, loss of less than one-third of the total area of meibomian glands (**B**); grade 2, loss of between one third and two-thirds of the total area (**C**), and grade 3, loss of over two-thirds of the total area (**D**).

Supplementary Table

Supplementary Table S1. Automatic calculation of parameters in meibomian glands at baseline in the analyzer

Number	Diameter(mm)	Length (mm)	Square (mm^2)	TI	SI
1	0.41	5.08	2.02	3.16	5.83
2	0.30	5.27	1.17	4.90	2.46
3	0.57	4.58	2.06	13.18	4.77
4	0.34	2.15	0.73	3.62	3.59
5	0.55	2.84	1.42	9.23	5.98
6	0.41	5.04	1.89	5.09	5.28
7	0.38	4.19	1.31	7.08	5.27
Average	0.42	4.17	1.51	6.61	4.74
	GA	59.22%			

Abbreviations: TI, tortuosity index; SI, signal index of the glands at baseline; GA, gland area

ratio at baseline.

Supplementary Table S2. Automatic calculation of parameters in meibomian glands at last visit in the analyzer

Number	Diameter(mm)	Length (mm)	Square (mm^2)	TI	SI
1	0.47	4.42	1.87	6.95	6.17
2	0.35	5.09	1.21	6.51	3.91
3	0.65	4.26	2.09	19.36	5.22
4	0.45	2.00	0.77	7.72	5.18
5	0.65	3.23	1.88	12.42	5.75
6	0.50	5.85	2.55	7.81	5.67
7	0.46	5.33	1.91	8.67	2.96
Average	0.50	4.31	1.75	9.92	4.98
	GA	67.02%			

Abbreviations: TI, tortuosity index; SI, signal index of the glands at baseline; GA, gland area ratio at baseline.

Supplementary Table S3. Univariable analysis examining the impact of relative variables on the percentage change of GA[a] (%)

Predictor	Unstandardized Coefficients Beta	95% CI		*P*
		Lower	Upper	
Age	-0.32	-0.47	-0.18	**<0.001**
Gender[c]	-10.06	-15.99	-4.12	**0.001**
TI[b]	-0.83	-1.46	-0.21	**0.010**
GA[b]	-0.53	-0.85	-0.20	**0.002**
SI[b]	-1.92	-3.66	-0.19	**0.031**
Onset duration	0.22	-0.53	0.97	0.553
Treatment duration	-0.11	-0.24	0.03	0.115

Abbreviations: GA, gland area ratio; TI, tortuosity index; SI, signal index of the glands. [a]: the percentage change of GA(%) = (GA at last visit - GA at baseline) / GA at baseline x 100.
[b]: value of indexes at baseline.[c]: male=0, female=1; set male as control. *P* values marked in **bold** indicate significance.
